# Systemic Expression, Purification, and Initial Structural Characterization of Bacteriophage T4 Proteins Without Known Structure Homologs

**DOI:** 10.3389/fmicb.2021.674415

**Published:** 2021-04-13

**Authors:** Kaining Zhang, Xiaojiao Li, Zhihao Wang, Guanglin Li, Biyun Ma, Huan Chen, Na Li, Huaiyu Yang, Yawen Wang, Bing Liu

**Affiliations:** ^1^BioBank, The First Affiliated Hospital of Xi’an Jiaotong University, Xi’an, China; ^2^Department of Life Sciences, Faculty of Natural Sciences, Imperial College London, London, United Kingdom; ^3^Department of Laboratory Medicine, The First Affiliated Hospital of Xi’an Jiaotong University, Xi’an, China; ^4^Department of Chemical Engineering, University of Loughborough, Leicestershire, United Kingdom

**Keywords:** bacteriophage, T4, NMR, cryo-EM, Mrh, Cef, Y04L and Gp57B

## Abstract

Bacteriophage T4 of *Escherichia coli* is one of the most studied phages. Research into it has led to numerous contributions to phage biology and biochemistry. Coding about 300 gene products, this double-stranded DNA virus is the best-understood model in phage study and modern genomics and proteomics. Ranging from viral RNA polymerase, commonly found in phages, to thymidylate synthase, whose mRNA requires eukaryotic-like self-splicing, its gene products provide a pool of fine examples for phage research. However, there are still up to 130 gene products that remain poorly characterized despite being one of the most-studied model phages. With the recent advancement of cryo-electron microscopy, we have a glimpse of the virion and the structural proteins that present in the final assembly. Unfortunately, proteins participating in other stages of phage development are absent. Here, we report our systemic analysis on 22 of these structurally uncharacterized proteins, of which none has a known homologous structure due to the low sequence homology to published structures and does not belong to the category of viral structural protein. Using NMR spectroscopy and cryo-EM, we provided a set of preliminary structural information for some of these proteins including NMR backbone assignment for Cef. Our findings pave the way for structural determination for the phage proteins, whose sequences are mainly conserved among phages. While this work provides the foundation for structural determinations of proteins like Gp57B, Cef, Y04L, and Mrh, other *in vitro* studies would also benefit from the high yield expression of these proteins.

## Introduction

With an estimated population of 10^31^, the bacteriophage—a type of virus preying on a bacterium—is the most abundant organism in the Earth’s biosphere (Clokie et al., 2011). It is believed that every bacterial strain hosts at least one type of phage, making the phage the most diversified organism (Keen, 2015). With such complexity, phage research has been focused on a few model phages that infect the most-studied bacteria like *Escherichia coli* and *Bacillus subtilis* (Miller et al., 2003). *Escherichia* virus T4 is one such example upon which studies have contributed to various aspects of viral biology since its discovery in the 1940s (Gamkrelidze and Dabrowska, 2014; Taj et al., 2014). As a member of the viral subfamily *Tevenvirinae*, T4 is also one of the seven known coliphages that specifically target *E. coli* and are lethal to cells (Yap and Rossmann, 2014). Along with other *E. coli* phages, it has provided instrumental tools for developing many fundamental biological concepts and applications. Some of the most significant discoveries in modern biology were aided by T4 phages, including the recognition of nucleic acids as genetic material, the demonstration that genetic codons are triplets, the discovery of mRNA, DNA restriction and modification, and self-splicing of intron/exon arrangements in prokaryotes (Kutter et al., 1995; Miller et al., 2003).

The double-stranded DNA genome of T4 is about 169 kbp in length, encoding about 289 proteins and bearing three eukaryotic-like introns (Comeau et al., 2007). T4-related phages exist in almost every ecosystem and represent a large portion of the tailed phages known to date (Amarillas et al., 2016). The functional annotations for the genes in these phages are mainly based on studies from T4, usually without any further verification. After years of effort, the majority of T4 gene products have been assigned to various functions, some of which have unique structures and features. Analysis of the sequential events during T4 infection has revealed the interactions between phage and the host, the strategies that phage employed to modulate host molecular machinery, as well as the specific function of individual proteins (Gamkrelidze and Dabrowska, 2014). For example, gene products of asiA have been characterized and determined to be anti-sigma factors in a wide range of phages (Sharma and Chatterji, 2008), including *Shigella* and *Acinetobacter* phages, thanks to studies into T4.

Although T4 is considered to be one of the best-known phages, approximately 130 out of its 289 proteins are still poorly described (Miller et al., 2003). For example, Y04L coded by the *y04L* gene in the pin-nrdC intergenic region is an example of proteins whose biological function and mechanism are yet to be addressed (Zhang et al., 2019). On the other hand, Cef is known to play a role in the maturation of viral tRNAs, a process that is still poorly characterized in phages (Pulitzer et al., 1985). Its molecular mechanism remains elusive, partially due to the absence of a relevant structure. Other examples include Mrh, which is a transcriptional regulator of late T4 genes that modulates the phosphorylation status of host heat shock sigma factor rpoH and thus promotes the attachment of host RNA polymerase to specific initiation sites (Mosig et al., 1998). The molecular basis remains unclear without structural interpretation on the Mrh protein and its binding partner. Thus, it is essential to study these poorly characterized proteins in order to further understand T4 phage biology. As phage study is gaining popularity due to the challenge from antibiotic-resistant bacteria (Lin et al., 2017), it is essential to assess the safety of phages in therapeutic applications. Without a fully annotated genome and functional interpretation of individual phage gene products, it is difficult to validate the safety of T4 related phages to be used in humans (Loc-Carrillo and Abedon, 2011).

This study attempts to express some of these proteins recombinantly in *E. coli* and in a cell-free system, as obtaining correctly folded proteins is the foundation for structure determination and *in vitro* functional analysis. We systemically screened the T4 genome and specifically looked for proteins with no homologous structure deposited in the Protein Data Bank (PDB). Finally, we selected 22 proteins for preliminary structural analysis. While some of these 22 proteins could be expressed as soluble proteins, others were found primarily in the inclusion body, possibly due to the toxicity to its native host *E. coli* or the lack of auxiliary T4 proteins that essential for proper folding. We applied two types of N-terminal fusion tags, SUMO and Msyb, to these proteins to improve the solubility and folding, with success in some cases (Marblestone et al., 2006; Yang et al., 2020). Using the Nuclear Magnetic Resonances (NMR) spectroscopy and Cryogenic electron microscopy, we provided an initial assessment for the folding and oligomeric states for soluble proteins and made progress on the structural determinations. Our systematic study provided preliminary data on expression conditions of the candidate proteins and shed light on the potential choice of techniques (solution NMR, X-ray crystallography, or Cryo-electron microscopy) for their structure determinations.

## Materials and Methods

### Plasmid Construction

The coding sequence of the candidate proteins was amplified from genomic DNA of the T4 phage using PCR. The PCR products were examined by agarose gel electrophoresis and further purified before being cloned into respective vectors to recombinantly attach the N-terminal His-tag (in-house modified pET-28 vector), His-SUMO tags (pET-SUMO vector), or His-Msyb tags (in-house modified from pET-SUMO), respectively. All primers and plasmids used are available in Supplementary Table 2. The resultant plasmids were transformed into *E. coli* DH5α cells. At least five independent clones of each plasmid were sequenced.

### Recombinant Protein Expression in *E. coli*

The expression vector was transformed into BL21(DE3) pLysS *E. coli* for protein expression. LB medium, supplemented with selected antibiotics (350 μg/mL of ampicillin, 485 μg/mL of kanamycin, or 378 μg/mL of carbenicillin) was used for the cultivation of *E. coli* strains. Minimal medium containing ^15^N-labeled ammonium chloride as the sole source of nitrogen and ^13^C-labeled glucose as the sole source of carbon were used to obtain uniformly ^15^N and ^13^C-enriched protein for structural determination using NMR.

The starting cultures were incubated overnight at 37°C with compatible antibiotics in a 50 ml centrifuge tube containing 30 ml of LB medium by inoculating a single colony from the plates. The starting cultures were then transferred into 1 L of fresh LB media or minimal media supplemented with the appropriate antibiotic. The cultures were incubating at 37°C until an optical density at 600 nm (OD_600_) of 0.6 was reached followed by the addition of 1 mM of IPTG to induce the expression. The cultures were then incubated overnight at 18°C before being harvested.

### Cell-Free Protein Expression

The coding sequence of Cef was coding optimized using the manufacturer’s online tool (Kangma-Healthcode, Shanghai). The *cef* gene was cloned into the cloned pD_2_P vector and amplified using DNA rolling circle amplification. The plasmid was then added into a 10 ml ProteinFactory fast reaction system. The reaction mixture was incubated for 3 h under room temperature with gentle shaking. The reaction mixture was then subjected to standard His-tagged protein purification or magnetic bead purification.

### Protein Purification

The culture was harvested by centrifugation at 5,000 rpm for 10 min at 4°C, resuspended in binding buffer (50 mM NaH_2_PO_4_, 300 mM NaCl, 10 mM imidazole, and pH 8.0), and lysed by sonication. Cell lysate was then centrifuged at 20,000 rpm for 30 min at 4°C. The supernatant or cell-free mixture was subjected to immobilized metal affinity chromatography (IMAC) and applied to the pre-equilibrated Ni-NTA resin (Qiagen). The resin was then washed with 5× wash buffer (50 mM NaH_2_PO_4_, 300 mM NaCl, 20 mM imidazole, and pH 8.0) before being eluted using an elution buffer (50 mM NaH_2_PO_4_, 300 mM NaCl, 250 mM imidazole, and pH 8.0) and dialyzed into respective buffers at 4°C overnight. The dialysis buffers used are detailed in Supplementary Table 3. Fractions were collected at each step, and aliquots were taken for Sodium dodecyl sulfate–polyacrylamide gel electrophoresis (SDS-PAGE). Cell debris was further resolubilized in denaturing buffer (8 M urea, 50 mM NaH_2_PO_4_, 300 mM NaCl, 10 mM imidazole, and pH 8.0), and an aliquot was taken for SDS-PAGE.

The SUMO tag was removed using SUMO protease, and the MysB tag was removed using TEV protease. The reaction mixtures were passed through the His-Trap column at least twice to remove the residual His-SUMO or His-Msyb.

### Size Exclusion Chromatography

Proteins were further purified and analyzed using an automated Äkta pure system in respective dialysis buffers. Different HiLoad columns were selected based on the molecular weight and the multimeric states of the proteins. Superdex 75 pg column was designed for proteins with smaller molecular weight (3–70 kDa), and Superdex 200 pg column was designed for protein polymers and proteins with large molecular weight (10–600 kDa).

### Sodium Dodecyl Sulfate–Polyacrylamide Gel Electrophoresis (SDS-PAGE)

The 12 μL protein samples were mixed with 3 μL 5× loading buffer and heated at 95°C for 5 min. The 10 μL mixtures were loaded in each lane of 20% polyacrylamide gels in the Mini Trans-Blot Cell system (BIO-RAD) running at 210 V for 1 h. Gels were stained with Coomassie brilliant blue for no less than 10 min followed by destaining in water overnight.

### Western Blot

Purified Y00G protein was loaded and separated by SDS-PAGE, and the gel was then transferred to the PVDF membrane. After blocking non-specific binding sites with 5% (w/v) BSA in buffer [10 mM Tris-HCl (pH 7.6), 100 mM NaCl, and 0.1% (v/v) Tween-20] for 2 h at room temperature (25°C), the primary antibody [His-tag antibody (2365S, CST)] was added overnight to the shaker at 4°C. Then, PVDF membranes were incubated with secondary antibodies (bs-40295G-HRP, Bioss, Beijing) for 1 h at room temperature. Western blot images were captured with the Amersham Imager 680 (GE).

### NMR Data Acquisition and Analysis

Size exclusion chromatography purified proteins were concentrated to at least 0.1 mM for 1D analysis and 0.5 mM for 2D and 3D NMR analysis. All NMR experiments were collected on a Bruker 600 MHz (Avance III) equipped with a cryoprobe. The water resonance was pre-saturated during the relaxation delay, and the chemical shift of the signal peak was determined with reference to D_2_O (4.72 ppm). The spectra were acquired using Topspin 10, processed using NmrPipe, and analyzed in NmrDraw. The phase and baseline of the spectra were corrected manually.

### Resonance Assignments for Cef

The near-complete backbone assignment was achieved by using NmrView5 with an in-house add-on script. Except for the first lysine and two proline residues, all of the 68 other crosspeaks in the 2D ^1^H–^15^N HSQC spectrum have been assigned. In total, 98% of all ^1^H, ^15^N, ^13^C’, ^13^Cα/β, 97% Hα, and 96% Hβ resonances were assigned. For sidechain resonances, 95.1% of aliphatic and aromatic sidechains (96.1% for H and 94.3% for C) were assigned, providing the basis for further structure determination.

### Cryo-EM Data Acquisition

The sample was diluted at a final concentration of around 0.3 mg/mL. A total of 3 mL of the sample were applied onto glow-discharged 200-mesh R2/1 Quantifoil copper grids. The grids were blotted for 4 s and rapidly cryocooled in liquid ethane using a Vitrobot Mark IV (Thermo Fisher Scientific) at 4°C and 100% humidity. The samples were imaged in a Titan Krios cryo-electron microscope (Thermo Fisher Scientific) operated at 300 kV with a GIF energy filter (Gatan) at a magnification of 105,000× (corresponding to a calibrated sampling of 0.82 Å per pixel). Micrographs were recorded by EPU software (Thermo Fisher Scientific) with a Gatan K3 Summit direct electron detector, where each image was composed of 30 individual frames with an exposure time of 2.5 s and an exposure rate of 22.3 electrons per second per Å^2^.

## Results

### Target Protein Selection

**FIGURE 1:**
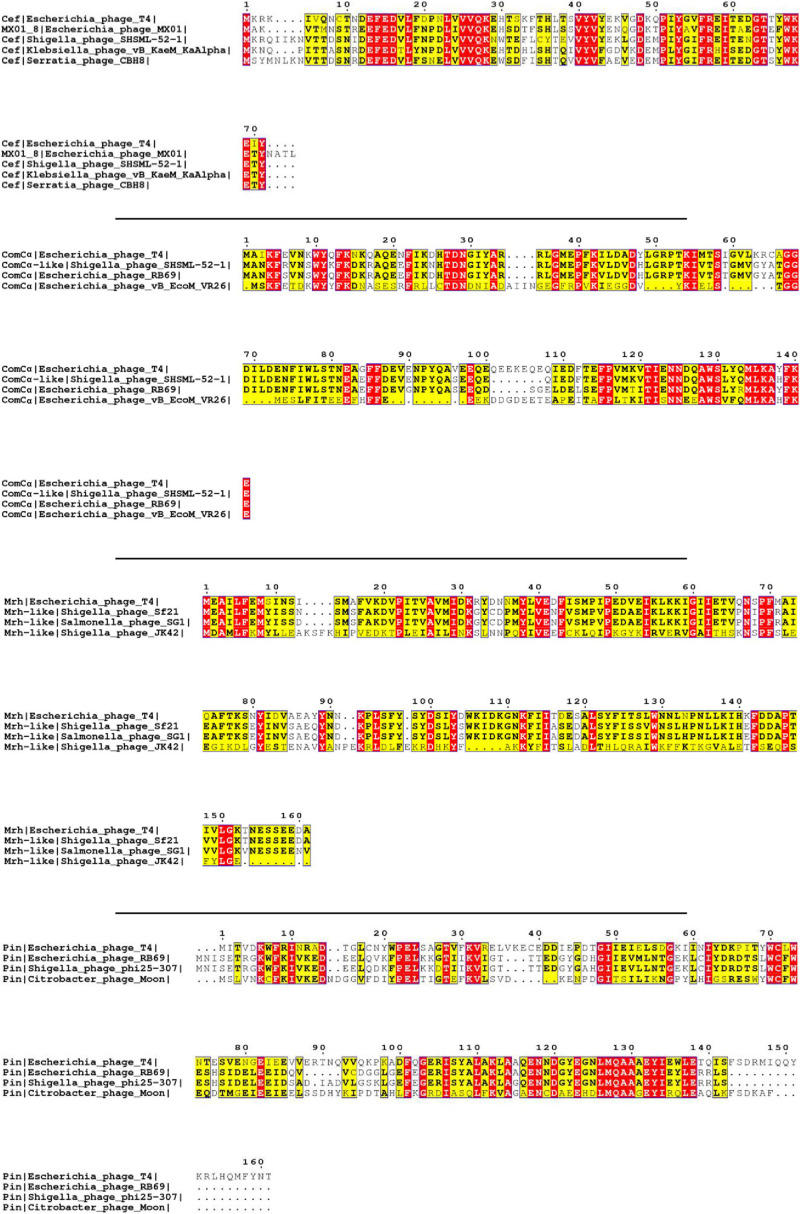
Multiple sequence alignment of selected T4 proteins. The multiple sequence alignments of Cef, ComCα, Mrh, and Pin of T4 showing the conserved regions of each viral protein with that of other phages. The purification and characterization of these proteins could provide a solid basis for future studies on these homologs of other phages. The red and yellow color is the visualization of “*,” which represents the fully conserved amino acid amongst all the sequences, and “:,” which represents amino acids with high similarities amongst the sequences (>0.5 in the Gonnet Pam 250 Matrix) used in the ClusterW alignment format.

The primary selection criterion for our proteins of interest is the absence of published homologous structure due to low sequence homology. On top of this, we filtered out small peptides with fewer than 50 amino acids in sequence, membrane proteins that may require bespoke protocols, and viral structural proteins that appear in the final virion assembly. After applying this filter, we selected 22 proteins from 268 reviewed T4 proteins listed in UniProt for our initial analysis (Table 1 and Supplementary Table 1). Using BLAST (Altschul et al., 1997) and Jpred (Cuff et al., 1998) for homology comparisons, we confirmed that the 22 proteins have no known structure homologs in PDB. In fact, all the amino acid sequences of these proteins are only conserved among bacteriophages, as seen from the multiple sequence alignments (Figure 1 and Supplementary Figure 1).

**TABLE 1:** Summary of the 22 proteins selected for the initial studies presented in this study, including their respective lengths and known functions.

Groups	Proteins	Length	Function
1	Y00H	81 aa	In dexA-dda intergenic region, function unknown
	Y00G	80 aa	Function unknown
	Y02D	125 aa	In imm-Gp43 intergenic region, function unknown
	Y00E	119 aa	In motB-dexA intergenic region, function unknown
	Y01A	103 aa	In dda-modA intergenic region, function unknown
	Y00F	166 aa	In motB-dexA intergenic region, function unknown
	Y04L	102 aa	In pin-nrdC intergenic region, function unknown (Zhang et al., 2019)
2	Cef	71 aa	Plays a role in the processing of a cluster of viral tRNAs (Pulitzer et al., 1985)
	Pin	161 aa	Plays a role in the inhibition of bacterial toxin-antitoxin system (Skorupski et al., 1988)
	MRH	161 aa	Plays a role in transcriptional regulation of late T4 genes (Mosig et al., 1998)
	DexA	227 aa	May play a role in the final step of host DNA degradation; Huang et al., 1999
	ComCα	141 aa	May act as a transcriptional antitermination factor (Stitt and Mosig, 1989)
	Motb	162 aa	Plays a role in the transcriptional regulation of middle promoters (Uzan et al., 1985)
	SegA	221 aa	Probably involved in the movement of the endonuclease-encoding DNA (Sharma and Hinton, 1994)
	segE	205 aa	Probably involved in the movement of the endonuclease-encoding DNA (Kadyrov et al., 1997)
	SegF	224 aa	Homing endonuclease (Belle et al., 2002)
	Gp57B	152 aa	Chaperones for tail fiber assembly include gp57A, gp57B, and Gp38 (Mosig and Eiserling, 2006)
	MobE	141 aa	Probable mobile endonuclease E (Wilson and Edgell, 2009)
3	RpbA	129 aa	Binds to host RNA polymerase core (Herendeen et al., 1990)
	alc	167 aa	Participates in the host transcription shutoff by causing premature termination of transcription from host DNA (Drivdahl and Kutter, 1990)
	Valyl	115 aa	Binds to the host valyl—tRNA ligase and thereby changes several of its physicochemical properties (Müller and Marchin, 1975)
	Gp64	274 aa	Binds to the viral DNA ends and protects the viral DNA against recBCD mediated degradation (Wang et al., 2000)

We then categorized the 22 proteins into three groups based on our current understanding (groups illustrated in Table 1). Y00H, Y00G, Y02D, Y00E, Y01A, Y00F, and Y04L fall into the first group, as none of its members have the function assigned. However, the genetic neighbors of these gene products may offer glimpses of their potential functions. For example, gene *y04L*, located in the pin-nrdC intergenic region, may have a role in blocking host Lon protease or phage ribonucleotide reducing reaction (Zhang et al., 2019). Y00E is another example of one whose neighboring genes are in the motB-dexA intergenic region. As the *dexA* gene (Gruber et al., 1988) codes for an exodeoxyribonuclease and the *motB* gene (Uzan et al., 1985) codes for a transcription regulatory protein, Y00E is likely to have a role in nucleic acids regulation.

The second category includes 11 proteins—Cef, Pin, MRH, DexA, ComCα, MotB, SegA, SegE, SegF, Gp57B, and MobE. These proteins are shown to participate in certain biological processes, and yet the exact binding partners or precise steps of the biological reaction involved remain inconclusive. For example, MRH was considered to play a role in regulating host heat-shock sigma factor RpoH and prevent phage progeny production in the ΔrpoH *E. coli* strain under high temperatures (Frazier and Mosig, 1990). ComCα was shown in regulating the expression of some T4 genes involved in DNA synthesis, such as the helicase Gp41, and was proposed to be a Rho-dependent transcriptional anti-termination factor. Cef (Stitt and Mosig, 1989) is responsible for the maturation of some of the phage tRNAs (Pulitzer et al., 1985), yet the exact function remains unclear.

The four remaining proteins fall in the last category. RpbA, alc, Valyl, and Gp64 are better understood among our selection, and their functions are assigned to specific events during phage invasion. Both RpbA and alc are involved in regulating host transcription—RpbA was shown to bind tightly to the *E.coli* RNA polymerase core (Herendeen et al., 1990), and protein alc is a site-specific transcription terminator that only inhibits transcriptional elongation on cytosine-containing DNA but not on the 5-hydroxymethyl cytosine present in the phage DNA (Kashlev et al., 1993). Valyl was shown to bind to host valyl-tRNA ligase and modifies its biochemical property (Müller and Marchin, 1977), and Gp64 is a DNA binding protein that binds to the termini of phage DNA, protecting it against host recBCD mediated degradation (Wang et al., 2000).

### His-Tagged Protein Expression and Purification

**FIGURE 2:**
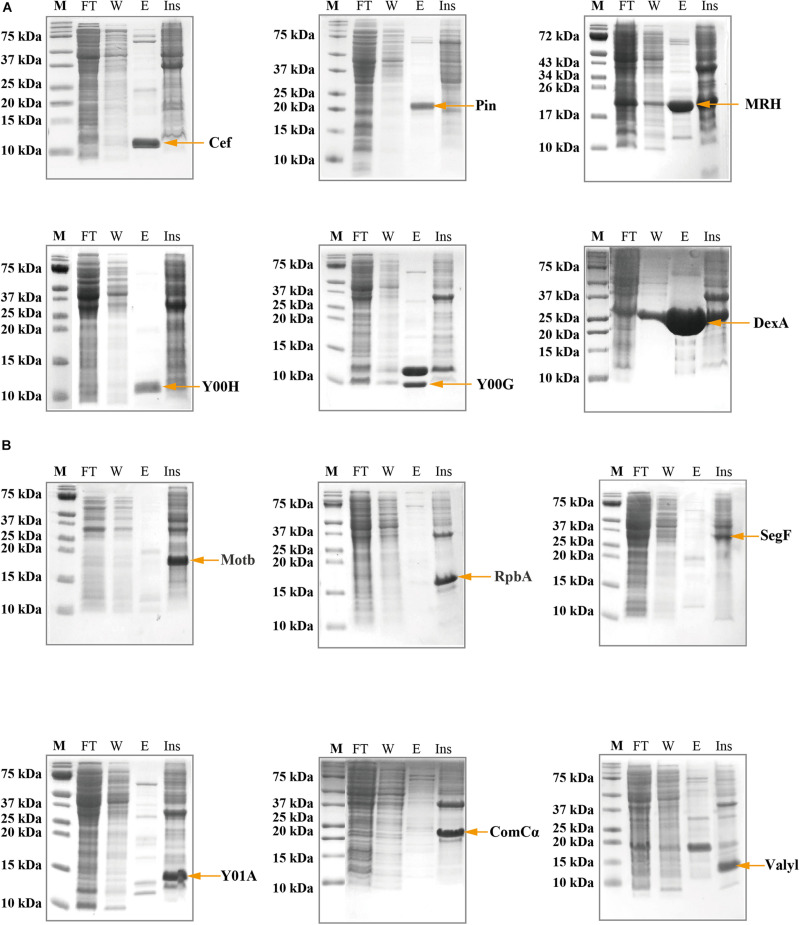
Sodium dodecyl sulfate–polyacrylamide gel electrophoresis (SDS-PAGE) showing the expression and the purification of the viral proteins using N-terminal His-tag. **(A)** Cef, Pin, MRH, Y00H, Y00G, and DexA proteins were collected in the soluble fraction, evident by the presence of bands of correct molecular weight in the elusion fraction. **(B)** MotB, RpbA, SegF, Y01A, ComCα, and Valyl proteins were expressed into the inclusion body, evident by the absence of bands of correct molecular weight in the elusion fraction and the presence of the bands in the insoluble fraction. M, Marker; FT, Flow-through fraction; W, Wash fraction; E, elusion fraction; Ins, Resolublized cell debris containing insoluble protein.

We subjected the 22 selected candidates to recombinant protein expression using the standard N-terminal His-tag. Expression trials were set up in different *E.coli* strains under various induction temperatures, times, and ITPG concentrations. To generalize the protocol, we chose the *E. coli* (pLyss) strain that was induced with 1mM IPTG and overnight induction at 18°C as the general protocol. In summary, MRH, Pin, MRH, Y00H, Y00G, and DexA had good yield in the soluble fraction (Figure 2A) while ComCα, RpbA, SegF, MotB, Valyl, SegE, MobE, alc, and Gp64 appeared in insoluble fraction (Figure 2B). Interestingly, two bands (approximately at 12 and 9 kDa) appeared in the gel of Y00G. Western blot using the anti-6X His tag antibody suggested that the 12 kDa band belonged to Y00G and the other might be an *E. coli* protein coeluted with the phage protein (Supplementary Figure 2). In contrast, Y02D, Y00E, Y04L, Gp57B, SegA, and Y00F had no success in recombination expression under the conditions we tested (data not shown). Thus, six proteins that could be expressed as soluble proteins were subjected to further purification and structural analysis. Furthermore, nine proteins that could be expressed as insoluble proteins were then tested for expression with protective tags and/or denature-refold preparation strategies (data not shown).

### Solubility Tag Expression: Sumo and Msyb

**FIGURE 3:**
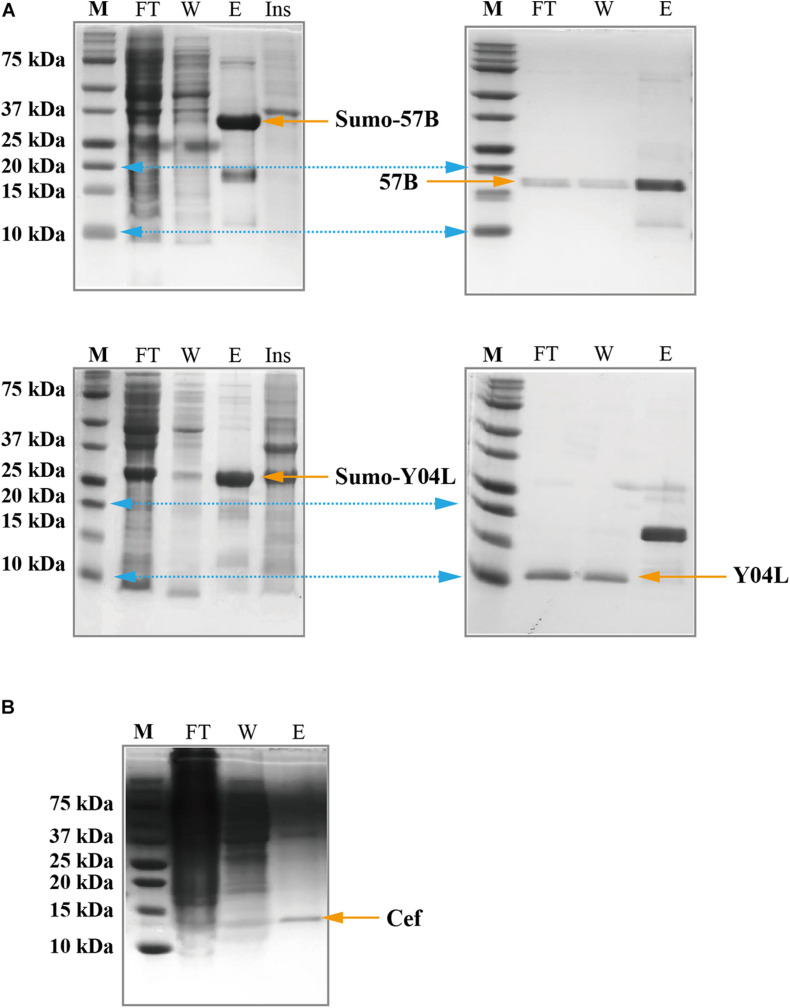
SDS-PAGE showing the expression and the purification of viral proteins using solubilization tags and the cell-free expression system. **(A)** SUMO-Gp57B and SUMO-Y04L were expressed and collected in the soluble fraction, evident by the presence of bands of correct molecular weight in the elusion fraction (left). The native proteins were collected after SUMO cleavage, evident by the bands in the flow-through and wash fraction (right). M, Marker; FT, Flow-through fraction; W, Wash fraction; E, Elusion fraction; Ins, Resolublized cell debris containing insoluble protein. **(B)** Cef protein was expressed and collected in the soluble fraction, using the cell-free expression system, evident by the presence of a sharp band at the corresponding size in the elution fraction. M, Marker; FT, Flow-through fraction; W, Wash fraction; E, Elusion fraction.

The SUMO tag fusion system can help recombinant proteins be expressed efficiently in *E. coli*, and SUMO can later be cleaved using SUMO-specific protease (Ulp1) to ensure the native activity of the target protein. The Msyb tag is a small acidic protein naturally found in *E.coli*, and it is another tag used to improve the solubility of the target protein (Yang et al., 2020). We constructed a plasmid (pET-Msyb) by replacing the gene encoding SUMO protein with the Msyb gene and a TEV cleavage site. As the SUMO tag can be removed without any extra residue left while TEV protease would leave an extra G, we chose the SUMO tag and presented our results. Strikingly, the yield of Gp57B and Y04L had improved considerably using the SUMO tag, and the fusion tag was removed using Ulp1 (Figure 3A).

### Cell-Free Expression

As *E. coli* is the native host of the T4 phage, recombination T4 proteins that are not expressed or appear in the inclusion body may have toxic effects and are thus guided away from the host’s soluble protein production (Ramón et al., 2014). To address this issue, we subjected the insoluble or unexpressed proteins (with or without fusion tags) to a yeast-based (*Kluyveromyces lactis*) cell-free expression system (Kangma-Healthcode, Shanghai). After codon optimization, the plasmid carrying the gene was added to the reaction mixture and harvested in 3 h under room temperature. Strikingly, Cef had reached a 5 mg yield in the 10 ml cell-free system (Figure 3B) and produced an almost identical spectrum as its counterparts produced in *E. coli* in ^1^H NMR analysis (Supplementary Figure 3).

### Size Exclusion Chromatography

**FIGURE 4:**
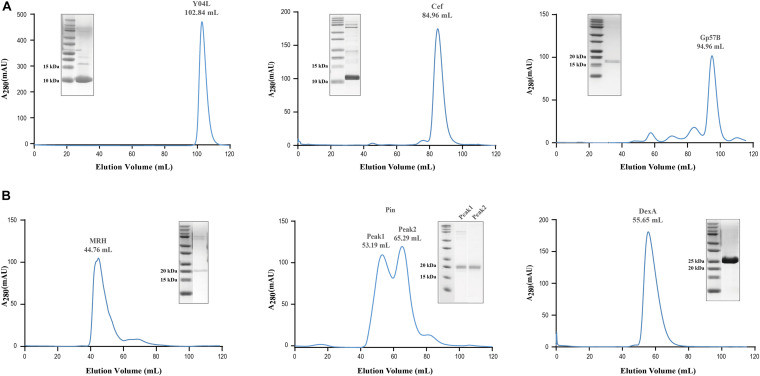
Size exclusion chromatography of soluble viral proteins for assessment of multimeric state in solution. **(A)** Size exclusion chromatography of Pin, Y04L, Cef, and Gp57B demonstrated the viral proteins existed as a homogenous monomeric state in solution, shown by the absorption peak at the volume of the expected molecular weight. **(B)** Size exclusion chromatography of MRH and DexA demonstrated a homogenous multimeric state in solution, as the proteins eluted at a lower volume, indicating a larger molecular weight. The chromatographies of Y04L and Gp57B were performed using HiLoad 16/600 Superdex 200 pg while the experiments of Cef, MRH, Pin, and DexA were using HiLoad 16/600 Superdex 75 pg.

The recombinantly expressed soluble proteins Pin, MRH, Y04L, DexA, Gp57B, and Cef underwent size exclusion chromatography to further purify and multimeric states analysis. While Pin, Y04L, Cef, and Gp57B appeared at expected elution volumes in the chromatograms (Figure 4A), DexA and MRH appeared at positions for protein with larger molecular weights (MW) (Figure 4B). Interestingly, the 25 kDa DexA likely exists as a dimer and the 19 kDa Mrh appears to be assembled into a large megadalton complex (Figure 4B). The results provide us with important information for further structural analysis and functional predictions of these proteins.

### Nuclear Magnetic Resonance Spectroscopy (NMR) Analysis

**FIGURE 5:**
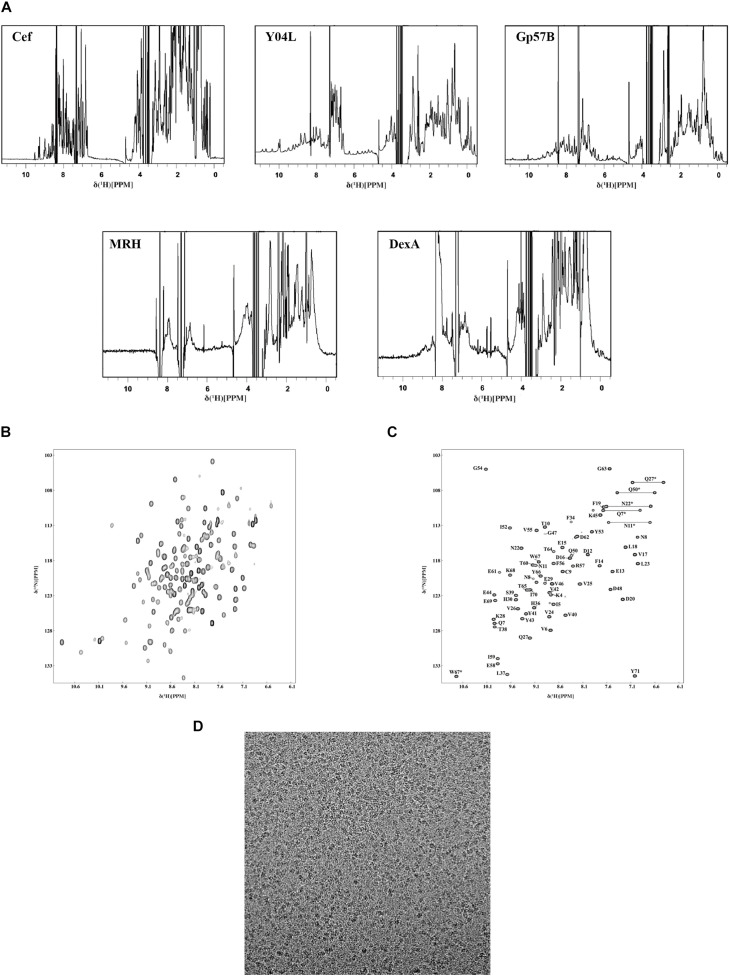
Characterization of the soluble phage proteins using NMR and Cryo-EM. **(A)** 1D ^1^H-NMR spectra of Cef, Y04L, and Gp57B showed characteristics of folded structural features, such as the presence of the sharp peaks below 1.0 ppm as well as the dispersion of peaks between 6.0 and 11.0 ppm. The ^1^H-NMR spectra of MRH and DexA showed the characteristics of a large multimeric protein, such as broad peak caused by the higher molecular weight, which was in agreement with the size exclusion chromatograms of MRH and DexA. **(B)** The 2D ^1^H-^15^N HSQC spectra of Y04L showed crosspeaks that were dispersed, indicating a folded structure in solution. **(C)** The assigned 2D ^1^H-^15^N HSQC spectra of Cef showed crosspeaks that were dispersed, indicating a folded structure in solution. The crosspeaks were assigned with the corresponding amino acid number and type. **(D)** The representative motion-corrected cryo-EM micrograph of DexA.

Using ^1^H NMR, we conducted an initial structural analysis of Cef, Y04L, Gp57B, MRH, and DexA. The spectra of Cef, Y04L, and Gp57B have good dispersion of peaks with upfield peaks around 0 ppm for aliphatic protons and downfield peaks around 10 ppm for amide protons, suggesting these protein are well-folded (Figure 5A). Meanwhile, MRH and DexA are in line with the characteristics of larger protein complexes in solution, with the former having characteristic dispersion of peaks for large protein and the latter has limited peaks in the 0 ppm region for folded protein. We then proceed to 2D ^1^H-^15^N HSQC experiments and structural determination for Cef, Y04L, and Gp57B. The HSQC spectra of all three proteins are as expected for the well-folded proteins of their respective MWs (Figure 5B). While we have demonstrated our work on Y04L (Zhang et al., 2019), the backbone assignments and structural calculations of Cef and Gp57B are currently in progress.

### Backbone Assignment of Cef

Using a standard multidimensional NMR technique, we started structural determination of Y04L and Cef. Except for the first two residues, four proline residues, all 96 other crosspeaks in the 2D ^1^H-^15^N HSQC spectrum have been assigned for Y04L (Zhang et al., 2019). For Cef, 68 residues, except for the first Methonine and two proline restudies, were assigned to the 2D ^1^H-^15^N HSQC spectrum (Figure 5C).

### Cryo-EM Single-Particle Analysis

To further understand the DexA and MRH complex, we performed cryogenic electron microscopy (cryo-EM) single-particle analysis of this complex obtained from size-exclusion chromatography. The samples produced optimal grids at a concentration of ∼0.5 mg/ml. We then solved the structure of MRH at 3.3 Å, which revealed a novel mechanism that the phage protein used to respond to heat shock (data not shown). While we continued the refinement of the structure of MRH, the images of DexA also show promising results, and the putative dimer is subjected to further structure analysis (Figure 5D).

## Discussion

Phages are very simple organisms. Of these, T4 is the most studied and has continuously provided discoveries for modern biology. Despite the numerous efforts since its discovery, 45% of T4 phage genes remain poorly characterized. Many of these genes could be functionally assigned using sequence homology comparison with the development of molecular and structural biology. For example, *segA* is predicted to code for an endonuclease that is probably involved in the movement of the endonuclease-encoding DNA, based on domain conservation. In contrast, the gene product of *mrh* was shown to play a role in transcriptional regulation of T4 late genes, yet its function remains elusive. Phage proteins like Mrh with no reported structure homologs are generally very poorly described even within this less characterized protein category. Our systemic work attempts to functionally assign some of these proteins through structural studies, thus complementing the current effects to annotate these genes in phage biology.

Using the conventional recombinant expression in *E. coli*, we found that a protein was coeluted with Y00G. Mass spectroscopy analysis suggested this protein belongs to *E. coli*, revealing the potential host binding partner of Y00G. In order to express soluble proteins for subsequent structural studies, the feasibility of using soluble tags and a cell-free expression system was explored in addition to routine His-tagged protein expression. SUMO and Msyb tags are relatively small tags that might facilitate a high yield of these small phage proteins without bringing a burden to the protein production system of *E. coli*. Indeed, proteins like Y04L benefitted greatly from the fusion tag with a high yield at 100 mg/L. Meanwhile, some phage proteins were still found in the inclusion body, and this is likely because these phage proteins are naturally interferers for *E. coli*. Thus, the cell-free system, yeast-based in particular, is ideal for expressing T4 proteins like RbpA, which interacts with *E. coli* RNA polymerase. We have demonstrated that Cef produced in the cell-free system had structural features identical to the one expressed in *E. coli*.

After the recombination proteins were crude purified using standard His-tag purification, size exclusion chromatography was applied for another purification and assessment of the potential multimeric states. Characterization tools, such as NMR or Cryo-EM, were utilized for the initial structural characterization of the proteins. Furthermore, proteins like Gp57B, Cef, and Y04L were subjected to structural determination by NMR, and the initial structures of Cef and Y04L (unpublished) suggested that these proteins adopt novel folds and are likely to have unique functions. Meanwhile, proteins like DexA and Mrh are in the structure determination using cryo-EM. These structures in the pipeline could provide molecular explanations for the observation in other biological experiments. Currently, structural prediction software relies heavily on the available structure templates, and the gene annotation of other phages is mainly relying on the understanding of proteins from model phages like T4 or λ. These structures, which do not have significant sequence homologs to published structures, would enrich the current pool of the protein structure database and provide templates for annotating other phage proteins.

## Data Availability Statement

The original contributions presented in the study are included in the article/Supplementary Material, further inquiries can be directed to the corresponding author/s.

## Author Contributions

KZ, XL, ZW, BM, HC, and NL performed the experiments. YW and BL designed the experiments. HY and BL analyzed the data. KZ, ZW, and BL wrote the manuscript. All authors contributed to the article and approved the submitted version.

## Conflict of Interest

The authors declare that the research was conducted in the absence of any commercial or financial relationships that could be construed as a potential conflict of interest.

## References

[B1] Amarillas L. Chaidez C. González-Robles A. León-Félix J. (2016). Complete genome sequence of new bacteriophage phiE142, which causes simultaneously lysis of multidrug-resistant *Escherichia coli* O157: H7 and *Salmonella enterica*. *Stand. Genomic Sci.* 11:89. 10.1186/s40793-016-0211-5 PMC5154165 27999624

[B2] Altschul S. F. Madden T. L. Schäffer A. A. Zhang J. Zhang Z. Miller W. (1997). Gapped BLAST and PSI-BLAST: a new generation of protein database search programs. *Nucleic Acids Res.* 25, 3389–3402. 10.1093/nar/25.17.3389 9254694 PMC146917

[B3] Belle A. Landthaler M. Shub D. A. (2002). Intronless homing: site-specific endonuclease SegF of bacteriophage T4 mediates localized marker exclusion analogous to homing endonucleases of group I introns. *Genes Dev.* 16 351–362. 10.1101/gad.960302 11825876 PMC155333

[B4] Clokie M. R. Millard A. D. Letarov A. V. Heaphy S. (2011). Phages in nature. *Bacteriophage* 1 31–45. 21687533 10.4161/bact.1.1.14942 PMC3109452

[B5] Comeau A. M. Bertrand C. Letarov A. Tétart F. Krisch H. (2007). Modular architecture of the T4 phage superfamily: a conserved core genome and a plastic periphery. *Virology* 362 384–396. 10.1016/j.virol.2006.12.031 17289101

[B6] Cuff J. A. Clamp M. E. Siddiqui A. S. Finlay M. Barton G. J. (1998). JPred: a consensus secondary structure prediction server. *Bioinformatics* 14 892–893. 10.1093/bioinformatics/14.10.892 9927721

[B7] Drivdahl R. H. Kutter E. M. (1990). Inhibition of transcription of cytosine-containing DNA in vitro by the alc gene product of bacteriophage T4. *J. Bacteriol.* 172 2716–2727. 10.1128/jb.172.5.2716-2727.1990 2185231 PMC208917

[B8] Frazier M. W. Mosig G. (1990). The bacteriophage T4 gene mrh whose product inhibits late T4 gene expression in an *Escherichia coli* rpoH (σ32) mutant. *Gene* 88 7–14. 10.1016/0378-1119(90)90053-t 1692800

[B9] Gamkrelidze M. Dabrowska K. (2014). T4 bacteriophage as a phage display platform. *Arch. Microbiol.* 196 473–479. 10.1007/s00203-014-0989-8 24828789 PMC4061479

[B10] Gruber H. Kern G. Gauss P. Gold L. (1988). Effect of DNA sequence and structure on nuclease activity of the DexA protein of bacteriophage T4. *J. Bacteriol.* 170 5830–5836. 10.1128/jb.170.12.5830-5836.1988 3056918 PMC211689

[B11] Herendeen D. R. Williams K. P. Kassavetis G. A. Geiduschek E. P. (1990). An RNA polymerase-binding protein that is required for communication between an enhancer and a promoter. *Science* 248 573–578. 10.1126/science.2185541 2185541

[B12] Huang Y.-J. Parker M. M. Belfort M. (1999). Role of exonucleolytic degradation in group I intron homing in phage T4. *Genetics* 153 1501–1512. 10581261 10.1093/genetics/153.4.1501 PMC1460841

[B13] Kadyrov F. A. Shlyapnikov M. G. Kryukov V. M. (1997). A phage T4 site-specific endonuclease, SegE, is responsible for a non-reciprocal genetic exchange between T-even-related phages. *FEBS Lett.* 415 75–80. 10.1016/s0014-5793(97)01098-3 9326373

[B14] Kashlev M. Nudler E. Goldfarb A. White T. Kutter E. (1993). Bacteriophage T4 Alc protein: a transcription termination factor sensing local modification of DNA. *Cell* 75 147–154. 10.1016/s0092-8674(05)80091-1 8402894

[B15] Keen E. C. (2015). A century of phage research: bacteriophages and the shaping of modern biology. *Bioessays* 37 6–9. 10.1002/bies.201400152 25521633 PMC4418462

[B16] Kutter E. Gachechiladze K. Poglazov A. Marusich E. Shneider M. Aronsson P. (1995). Evolution of T4-related phages. *Virus Genes* 11 285–297. 10.1007/bf01728666 8828153

[B17] Lin D. M. Koskella B. Lin H. C. (2017). Phage therapy: an alternative to antibiotics in the age of multi-drug resistance. *World J. Gastrointest. Pharmacol. Ther.* 8:162. 10.4292/wjgpt.v8.i3.162 28828194 PMC5547374

[B18] Loc-Carrillo C. Abedon S. T. (2011). Pros and cons of phage therapy. *Bacteriophage* 1 111–114. 10.4161/bact.1.2.14590 22334867 PMC3278648

[B19] Marblestone J. G. Edavettal S. C. Lim Y. Lim P. Zuo X. Butt T. R. (2006). Comparison of SUMO fusion technology with traditional gene fusion systems: enhanced expression and solubility with SUMO. *Protein Sci.* 15 182–189. 10.1110/ps.051812706 16322573 PMC2242369

[B20] Miller E. S. Kutter E. Mosig G. Arisaka F. Kunisawa T. Ruger W. (2003). Bacteriophage T4 genome. *Microbiol. Mol. Biol. Rev.* 67 86–156. 12626685 10.1128/MMBR.67.1.86-156.2003 PMC150520

[B21] Mosig G. Eiserling F. (2006). “T4 and related phages: structure and development,” in *The Bacteriophages*, 2nd Edn, ed. Calendar R. (Oxford: Oxford University Press), 225–267.

[B22] Mosig G. Colowick N. E. Pietz B. C. (1998). Several new bacteriophage T4 genes, mapped by sequencing deletion endpoints between genes 56 (dCTPase) and dda (a DNA-dependent ATPase-helicase) modulate transcription. *Gene* 223 143–155. 10.1016/s0378-1119(98)00238-8 9858714

[B23] Müller U. Marchin G. (1977). Purification and properties of a T4 bacteriophage factor that modifies valyl-tRNA synthetase of *Escherichia coli*. *J. Biol. Chem.* 252 6640–6645. 10.1016/s0021-9258(17)39895-2 19475

[B24] Müller U. Marchin G. L. (1975). Temporal appearance of bacteriophage T4-modified valyl tRNA synthetase in *Escherichia coli*. *J. Virol.* 15 238–243. 10.1128/jvi.15.2.238-243.1975 163351 PMC354445

[B25] Pulitzer J. F. Colombo M. Ciaramella M. (1985). New control elements of bacteriophage T4 pre-replicative transcription. *J. Mol. Biol.* 182 249–263. 10.1016/0022-2836(85)90343-2 3999145

[B26] Ramón A. Señorale M. Marín M. J. F. I. M. (2014). Inclusion bodies: not that bad…. *Front. Microbiol.* 5:56. 10.3389/fmicb.2014.00056 24592259 PMC3924032

[B27] Sharma M. Hinton D. M. (1994). Purification and characterization of the SegA protein of bacteriophage T4, an endonuclease related to proteins encoded by group I introns. *J. Bacteriol.* 176 6439–6448. 10.1128/jb.176.21.6439-6448.1994 7961394 PMC196996

[B28] Sharma U. K. Chatterji D. (2008). Differential mechanisms of binding of anti-sigma factors *Escherichia coli* Rsd and bacteriophage T4 AsiA to E. coli RNA polymerase lead to diverse physiological consequences. *J. Bacteriol.* 190 3434–3443. 10.1128/jb.01792-07 18359804 PMC2394999

[B29] Skorupski K. Tomaschewski J. Rüger W. Simon L. (1988). A bacteriophage T4 gene which functions to inhibit *Escherichia coli* Lon protease. *J. Bacteriol.* 170 3016–3024. 10.1128/jb.170.7.3016-3024.1988 2838455 PMC211243

[B30] Stitt B. L. Mosig G. (1989). Impaired expression of certain prereplicative bacteriophage T4 genes explains impaired T4 DNA synthesis in *Escherichia coli* rho (nusD) mutants. *J. Bacteriol.* 171 3872–3880. 10.1128/jb.171.7.3872-3880.1989 2544560 PMC210138

[B31] Taj M. K. Samreen Z. Taj I. Hassani T. M. Ling J. Yunlin W. (2014). T4 bacteriophage as a model organism. *IMPACT Int. J. Res. Appl. Nat. Soc. Sci.* 2 19–24.

[B32] Uzan M. d’Aubenton-Carafa Y. Favre R. de Franciscis V. Brody E. (1985). The T4 mot protein functions as part of a pre-replicative DNA-protein complex. *J. Biol. Chem.* 260 633–639. 10.1016/s0021-9258(18)89779-4 3880747

[B33] Wang G. Vianelli A. Goldberg E. (2000). Bacteriophage T4 self-assembly: in vitro reconstitution of recombinant gp2 into infectious phage. *J. Bacteriol.* 182 672–679. 10.1128/jb.182.3.672-679.2000 10633100 PMC94329

[B34] Wilson G. W. Edgell D. R. (2009). Phage T4 mobE promotes trans homing of the defunct homing endonuclease I-TevIII. *Nucleic Acids Res.* 37 7110–7123. 10.1093/nar/gkp769 19773422 PMC2790892

[B35] Yang Y. Ke Z. Wang Z. Li Y. Li Y. Wang Y. (2020). 1H, 13C and 15 N NMR assignments of solubility tag protein Msyb of *Escherichia coli*. *Biomol. NMR Assign.* 14 251–254. 10.1007/s12104-020-09955-6 32504338

[B36] Yap M. L. Rossmann M. G. (2014). Structure and function of bacteriophage T4. *Future Microbiol.* 9 1319–1327. 10.2217/fmb.14.91 25517898 PMC4275845

[B37] Zhang K. Wang Z. Chang G. Wang H. Wang Y. Liu B. (2019). Resonance assignments of bacteriophage T4 Y04L protein. *Biomol. NMR Assign.* 14 51–54. 10.1007/s12104-019-09919-5 31707562

