# Supplementary material for: Systemic Expression, Purification, and Initial Structural Characterization of Bacteriophage T4 Proteins Without Known Structure Homologs
Source: Front Microbiol. 2021 Apr 13;12:674415. doi: 10.3389/fmicb.2021.674415 (PMC8076793; doi:10.3389/fmicb.2021.674415)
Supplement: Supplementary file 1 [file Data_Sheet_1.PDF]

Supplementary Figure 1: Multiple sequence alignment of T4 proteins. The multiple sequence alignments of the remaining 18 T4 viral proteins showing the conserved regions of each viral protein with that of other phages. The purification and characterization of these proteins could provide a solid basis for future studies on these homologues of other phages. The red and yellow colour is the visualization of "*" which represents the fully conserved amino acid amongst all the sequence, and ":" which represents amino acid with high similarities amongst the sequences (>0.5 in the Gonnet Pam 250 Matrix) used in the Cluster Walignment format.

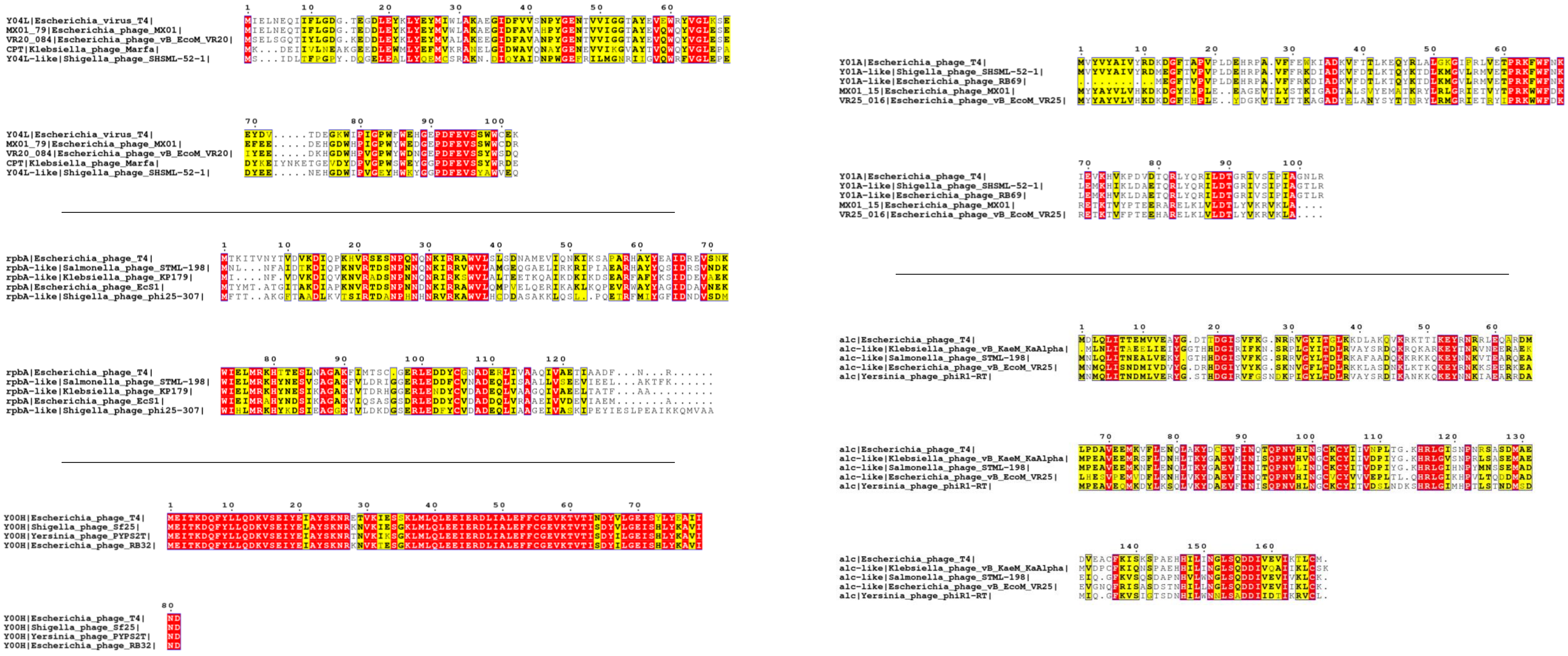

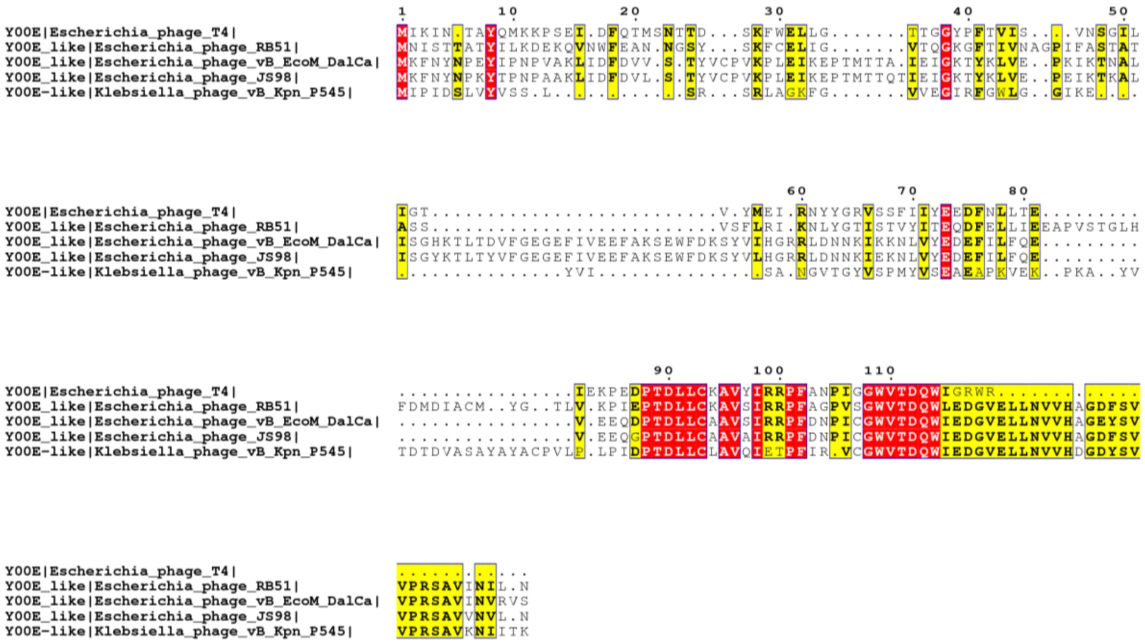
Y00E|Escherichia_phage_T4|
Y00E_like|Escherichia_phage_RB51|
Y00E_like|Escherichia_phage_vB_EcoM_DalCa|
Y00E_like|Escherichia_phage_JS98|
Y00E-like|Klebsiella_phage_vB_Kpn_P545|

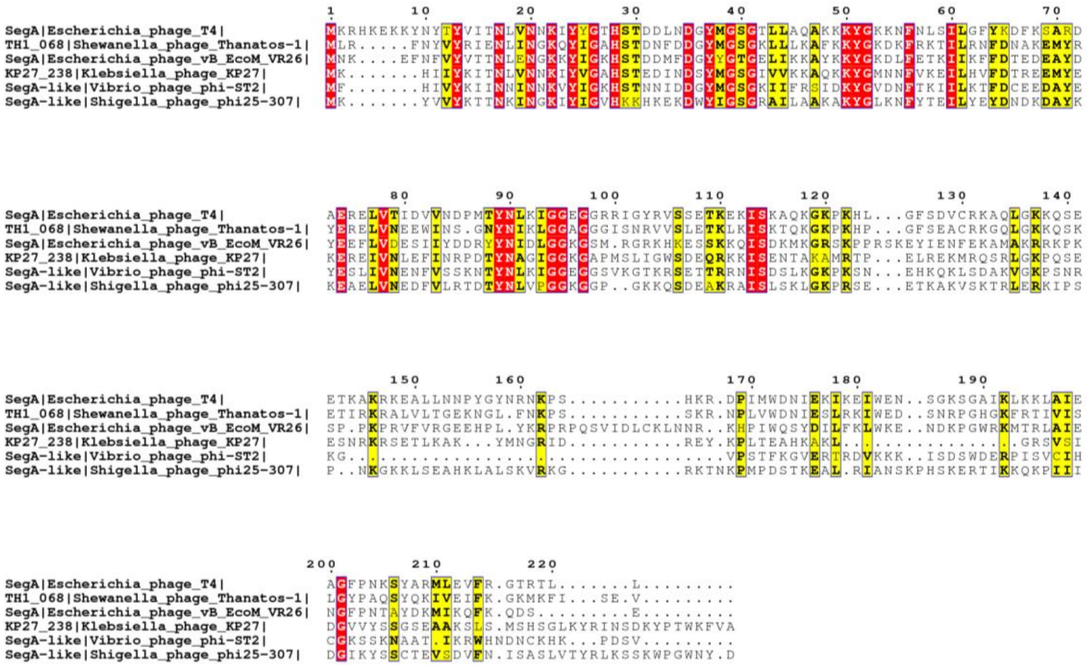
SegA|Escherichia_phage_T4|
TH1_068|Shewanella_phage_Thanatos-1|
SegA|Escherichia_phage_vB_EcoM_VR26|
KP27_238|Klebsiella_phage_KP27|
SegA-like|Vibrio_phage_phi-ST2|
SegA-like|Shigella_phage_phi25-307|

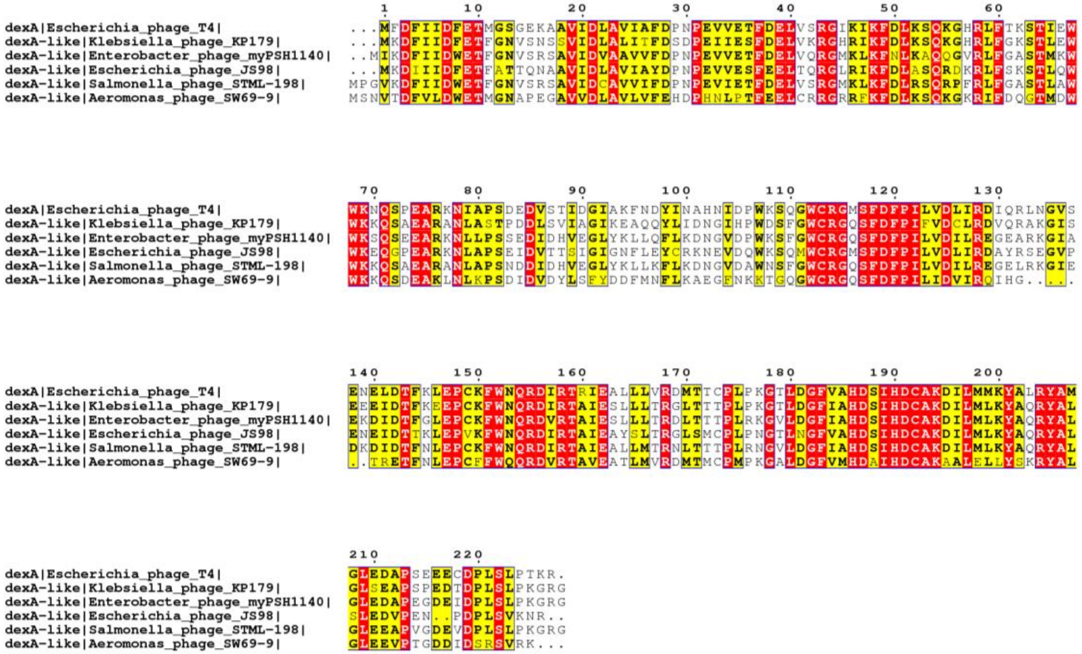
dexA|Escherichia_phage_T4|
dexA-like|Klebsiella_phage_KP179|
dexA-like|Enterobacter_phage_myPSH1140|
dexA-like|Escherichia_phage_JS98|
dexA-like|Salmonella_phage_STML-198|
dexA-like|Aeromonas_phage_SW69-9|

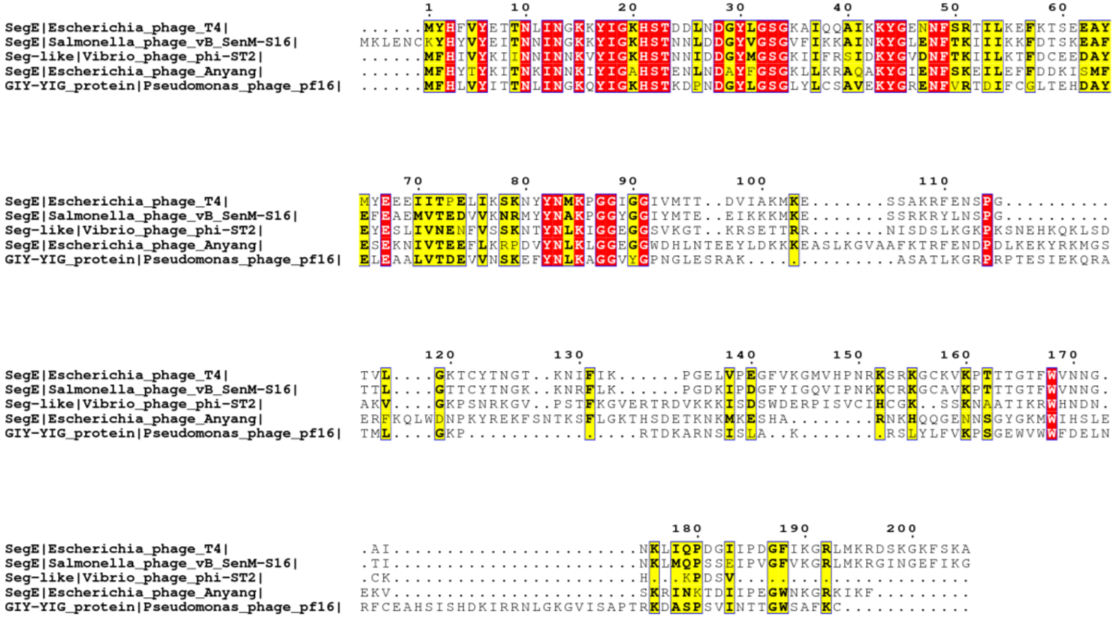
SegE|Escherichia_phage_T4|
SegE|Salmonella_phage_vB_SenM-S16|
Seg-like|Vibrio_phage_phi-ST2|
SegE|Escherichia_phage_Anyang|
GIY-YIG_protein|Pseudomonas_phage_pf16|

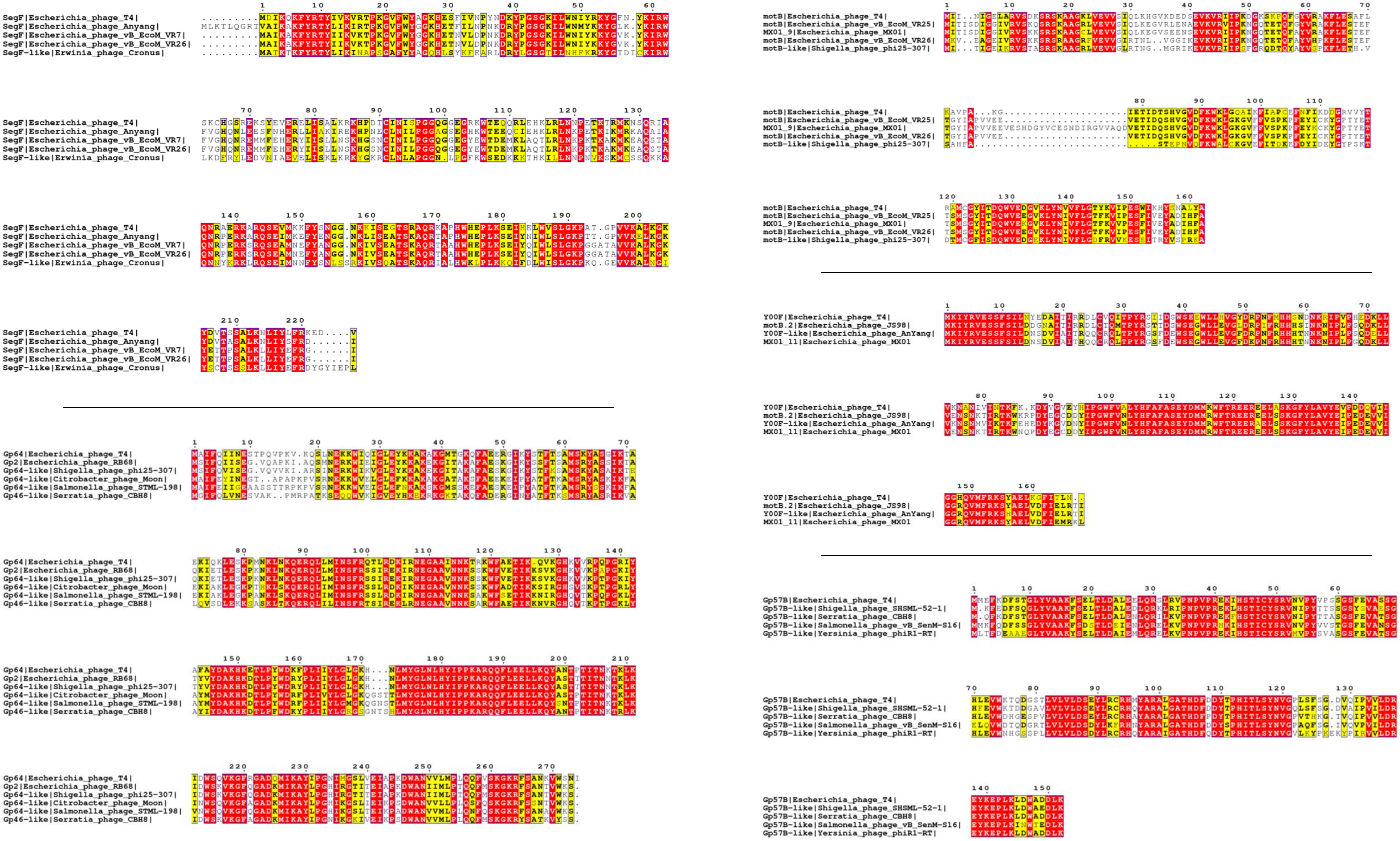
SegF|Escherichia_phage_T4|
SegF|Escherichia_phage_Anyang|
SegF|Escherichia_phage_vB_EcoM_VR7|
SegF|Escherichia_phage_vB_EcoM_VR26|
SegF-like|Erwinia_phage_Cronus|
Gp64|Escherichia_phage_T4|
Gp2|Escherichia_phage_RB68|
Gp64-like|Shigella_phage_phi25-307|
Gp64-like|Citrobacter_phage_Moon|
Gp64-like|Salmonella_phage_STML-198|
Gp46-like|Serratia_phage_CBH8|
motB|Escherichia_phage_T4|
motB|Escherichia_phage_vB_EcoM_VR25|
MX01_9|Escherichia_phage_MX01|
motB|Escherichia_phage_vB_EcoM_VR26|
motB-like|Shigella_phage_phi25-307|
Y00F|Escherichia_phage_T4|
motB.2|Escherichia_phage_JS98|
Y00F-like|Escherichia_phage_AnYang|
MX01_11|Escherichia_phage_MX01
Gp57B|Escherichia_phage_T4|
Gp57B-like|Shigella_phage_SHSML-52-1|
Gp57B-like|Serratia_phage_CBH8|
Gp57B-like|Salmonella_phage_vB_SenM-S16|
Gp57B-like|Yersinia_phage_phiR1-RT|

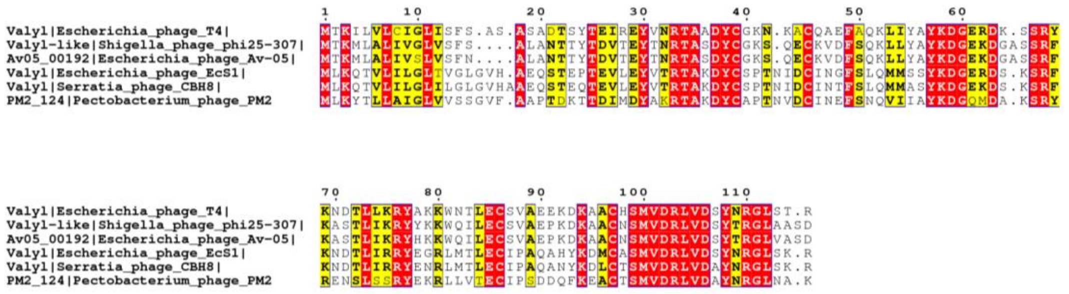
Valyl|Escherichia_phage_T4|
Valyl-like|Shigella_phage_phi25-307|
Av05_00192|Escherichia_phage_Av-05|
Valyl|Escherichia_phage_EcS1|
Valyl|Serratia_phage_CBH8|
PM2_124|Pectobacterium_phage_PM2

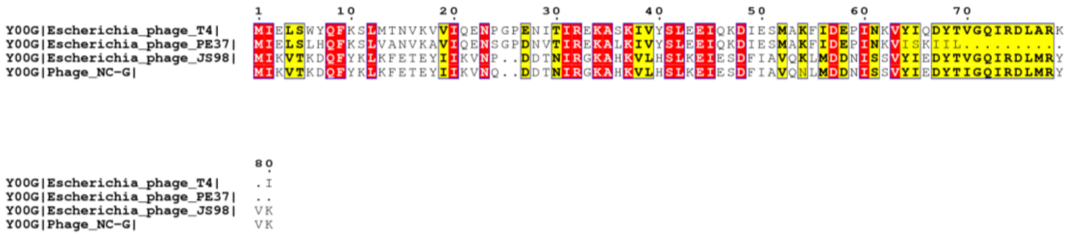
Y00G|Escherichia_phage_T4|
Y00G|Escherichia_phage_PE37|
Y00G|Escherichia_phage_JS98|
Y00G|Phage_NC-G|

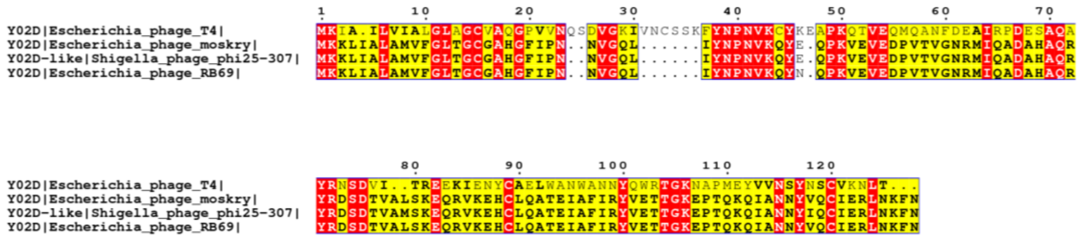
Y02D|Escherichia_phage_T4|
Y02D|Escherichia_phage_moskry|
Y02D-like|Shigella_phage_phi25-307|
Y02D|Escherichia_phage_RB69|

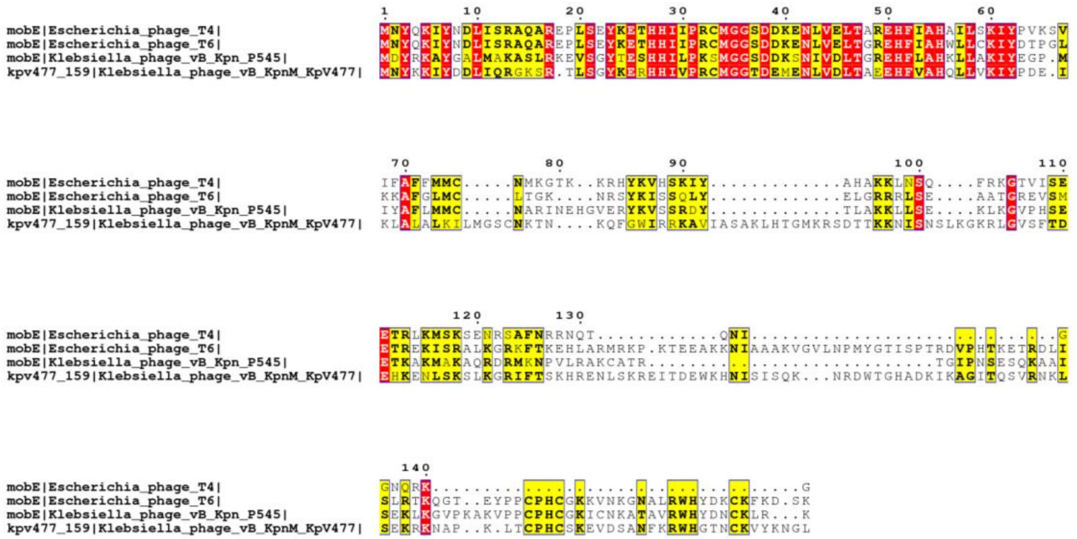
mobE|Escherichia_phage_T4|
mobE|Escherichia_phage_T6|
mobE|Klebsiella_phage_vB_Kpn_P545|
kpv477_159|Klebsiella_phage_vB_KpnM_KpV477|

Supplementary Figure 2: Western blot image of Y00G.

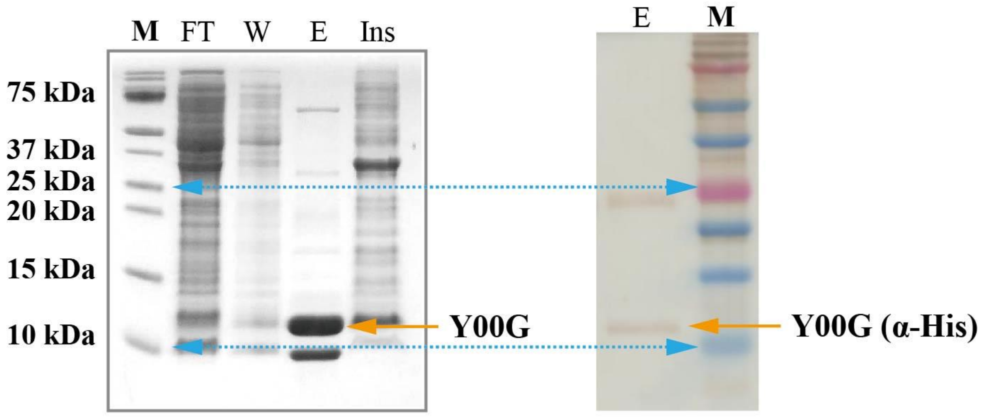

Supplementary Figure 3: ^{1}H NMR spectra overlay of Cell-free and *E. coli* produced Cef.

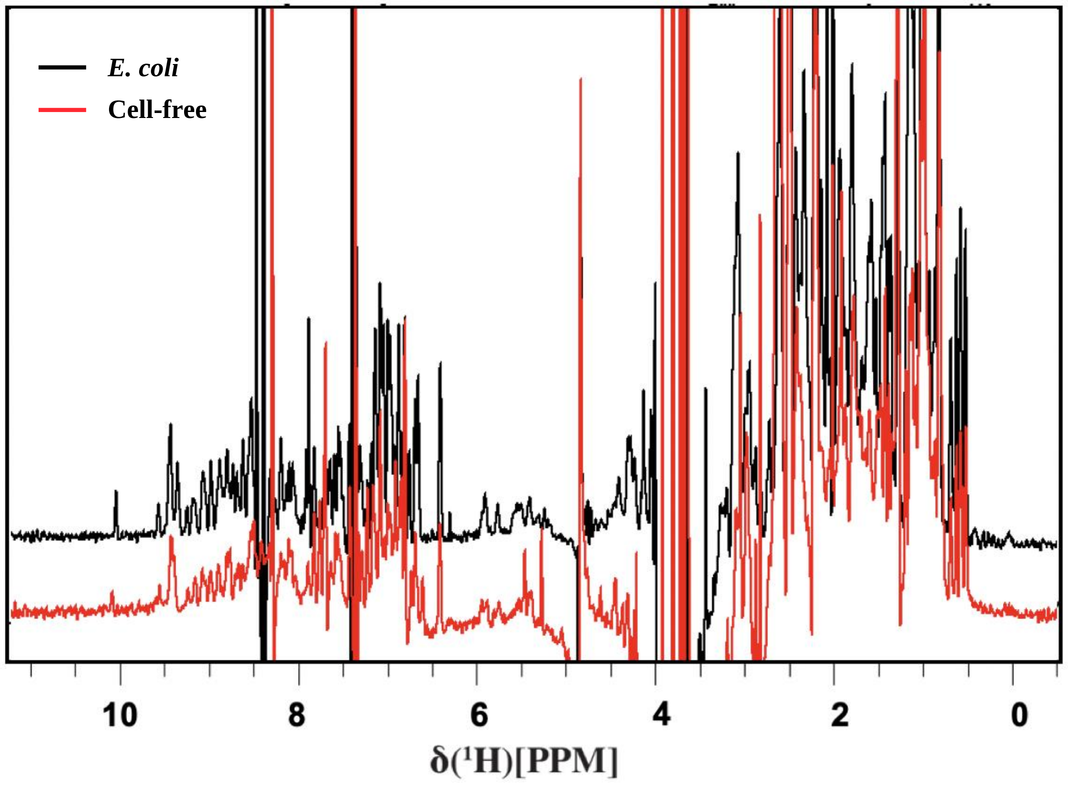

Supplementary Table 1
UniProt entry number

Protein	UniProt entry number
Y00H	P39418
Y00G	P32285
Y02D	P18057
Y00E	P39415
Y01A	P39419
Y00F	P39416
Y04L	P07070
Cef	Q01436
Pin	P07068
MRH	P16007
DexA	P04536
ComCα	Q01438
Motb	Q01437
SegA	P32286
segE	P18060
SegF	D9IE60
Gp57B	P04533
MobE	P32283
RpbA	P07879
alc	P04546
Valyl	P13310
Gp64	P15076

Supplementary Table 2
Primer list

pET28a-Valyl	Sense: CACCATGACTAAAATTTTGGTTTTATGTATAGGATTAATTTCA
	Antisense: TTATCATCTAGTACTCAATCCTCGATTATAAGAAT
pET28a-57B	Sense: CACCATGATGGAATTTAAAGACTTTTCAACGG
	Antisense: TTATTATTTAAATCATCTGCCCAATCGAGTT
pET28a-MobE	Sense: CACCATGAATTATCAAAAAATCTATAACGACCTAATTTCC
	Antisense: TTATTAGCCCTTCCTTTGGTTGC
pET28a-gp49.3	Sense: CACCATGATAGAATTAAATGAACAAATTATTTTTCTAGGCG
	Antisense: TTATCATTTTTCACACCACCAACTGC
pET28a-Terminal DNA	Sense: CACCATGGCTATTTTTCAAATAATTAATGAAAGCACT
	Antisense: TTATTAGATATTTGACCAGACTTTGTTTGCA
pET28a-SegE	Sense: CACCATGTACCACTTTGTATATGAAACAACAAATC
	Antisense: TTATTATGCCTTACTAAATTTGCCTTTAGAATCT
pET28a-alc	Sense: CACCATGGATTTACAACTTATTACTACTGAAATGGT
	Antisense: TTATTACATGCATAAAGTTTTAATAACCTCTACAATATC
pET28a-Pin	Sense: CACCATGATTACAGTAGATAAATGG
	Antisense: TTAAGTATTGTAAAACATTTGATGC
pET28a-Y00G	Sense: CACCATGATTGAATTAAGTTGGT
	Antisense: TTAAATTTTCCTCGCTAAATCA
pET28a-Y00H	Sense: CACCGTGGAAATAACTAAAGATCAG
	Antisense: TTAATCATTAATAATCGCCTCA
pET28a-Cef	Sense: CACCATGAAACGTAAAATTGTTCAG
	Antisense: TTAATAAATTTCCTTCCAGT
pET28a-DexA	Sense: CACCATGTTTGATTTTATTATAGATTTTGAAAC
	Antisense: TTATCGTTTTGTTGGAAGAGATAG
pET28a-Motb	Sense: CACCATGATTATTAATATTGGTG
	Antisense: TTATGCATAGAGAGCATTGC
pET28a-SegA	Sense: CACCATGAAAAGACATAAAGAAAAG
	Antisense: TTATAGTAGTGTTCTTGTTCCACG
pET28a-SegF	Sense: CACCATGGATATTAAACAAAAATTTTATAGAAC
	Antisense: TTATACATCTTCTTTTCTGAATAAG
pET28a-RpbA	Sense: CACCATGACTAAAATTACTGTGAATTATACTG
	Antisense: TTAACGATTAAAATCAGCCGC
pET28a-ComCα	Sense: CACCATGGCTATTAAATTTGAAGTTAATAAATG
	Antisense: TTATTCCTTAAAGTAAGCTTTC
	Sense: CACCATGAAAATCGCTATTTTGGTTATTG

pET28a-Y02D	Antisense: TTAGGTCAAATTTTTTAC
pET28a-Y00E	Sense: CACCATGATTAAAATTAATACTGCG
	Antisense: TCAACGCCATCTTCCAATCC
pET28a-Y00F	Sense: CACCATGAAAATTTATCGTGTTGAATCATCGTTTAG
	Antisense: CGAACTAAAAGGCTTTATTACCTTAAACTAA
pET28a-mRH	Sense: ATGGAAGCAATTTTGTTTGAAATG
	Antisense: TTATCAAGCATCTTCTTCAGAACTTTC
pET-SUMO-gp57B	Sense: GAACAGATTGGTGGTATGATGGAATTTAAAGACTTTTCAACGGG
	Antisense: GTGGTGCTCGAGCTATTATTTTAAATCATCTGCCCAATCGAGTT
pET-SUMO-gp11.9	Sense: GAACAGATTGGTGGTATGATAGAATTAAATGAACAAATTATTTTTC TAGGCG
	Antisense: GTGGTGCTCGAGCTATCATTTTTCACACCACCAACTGC
pET-Mysb-y01A	Sense: CTTTATTTTCAGGGCATGGTTTATGTATATGCGATAGTTTACCG
	Antisense: GTGGTGCTCGAGCTATCATCGTAAATTCCCTGCAATTGG

Supplementary Table 3
Dialysis Buffers

DexA	250mM NaCl, 50mM NaH_2PO_4, 0.2mM TCEP, pH 6.5
MRH	
Gp11.9	100mM NaCl, 50mM K_2HPO_4, 5mM EDTA, 2mM DTT, pH 5.5
Gp57B	250mM NaCl, 50mM Tris, 2mM DTT, pH 6.5
Cef	250mM NaCl, 50mM Tris, 0.2mM TCEP, pH 7.0
Pin	250mM NaCl, 50mM Tris, 0.2mM TCEP, pH 7.5
Y00G	
Y00H	
